# Discussion on Fevers and Other Subjects, by Austin District Medical Society

**Published:** 1892-01

**Authors:** H. A. West

**Affiliations:** Theory and Practice Medicine, Texas Medical College


### Society Notes.

### AUSTIN DISTRICT MEDICAL SOCIETY, MEETING DEC. 17, 1891.—REPORT OF PROCEEDINGS CONTINUED.

### THE SUBJECT OF FEVERS IN TEXAS DISCUSSED BY PROF. H. A. WEST, M. D., THEORY AND PRACTICE MEDICINE, TEXAS MEDICAL COLLEGE.

Dr. H. A. West, of Galveston, at the request of the Society, proceeded to exhibit some interesting pathological specimens, with the following explanatory remarks:

The first specimen shown is a heart, illustrating an extreme degree of calcarious degeneration of the aortic and mitral valves, and hypertrophy of the left ventricle. The case was an interesting one from several points of view. The patient, H. L., a native of Germany; age 72 years; admitted to the Sealy hospital Nov. 9; dyspnœa, orthopnæ and cyanosis noted, also extreme restlessness, inability to sleep, and disposition to talk and wander about the ward.

Physical examination of the lungs revealed sonorous and sibilant rales diffused over the chest. The apex-beat of the heart was displaced to the left; upon auscultation a loud double murmur was heard all over the cardiac area. At the base the bruit was diastolic, very loud and rasping; there was a softer systolic murmur at the apex. Upon elevation of the arm the pulsus celer was noted. Ten drop doses tinct. digitalis was ordered three times daily. Attention was called to the possible danger of opiates in such cases. The patient became so noisy and disturbing to the other patients, that the resident physician was induced to administer a hypodermic injection of morphia and atropia. Patient died Nov. 14th, the end no doubt being hastened by the opiate.

The autopsy revealed the condition here shown, viz: calcification of the cusps of the aortic valve, the ensuing stiffening and contraction preventing perfect closure, and at the same time narrowing the opening at the base of the valves. Surrounding the entrance to the aorta is a dense ring of calcified tissue, a quarter of an inch in thickness, and apparently as hard as bone; the mitral valves showed similar lesions, but not so well marked. The walls of the left ventricle were enormously thickened.

Mention was made of an interesting fact in connection with the case, viz: About ten years ago the patient had applied for insurance in one of the endowment organizations, and was rejected on account of this heart lesion. How long previously the condition had been coming on, is not known. The case is a beautiful illustration of nature's conservatism in compensating by ventricular hypertrophy and allowing the patient's life to continue for so long a time in the presence of such a lesion as this.

The next two specimens were sections of the ilium and cæcum, showing the lesions of enteric fever, induration and thickening of Peyer's patches, and numerous round and oval ulcers, especially pronounced nearer the cæcum; some of these ulcers involving only the mucous and submucous membranes, others the muscular coat, and one perforating the serosa.

The long diameter of these ulcers was transverse to the bowel, contrary to what is said to be the usual position. There were no ulcers in the colon; the spleen in both cases was enlarged. Also the mesenteric glands in the lungs of one, were evidences of old healed tubucular deposits.

There had been twelve cases of enteric fever admitted to the Sealy hospital since 1st Oct., 1891. Clinical histories and charts are here presented of them. About one-half the number were from various points in the interior of Texas; several had come from the rock quarries in Washington county, near Gay Hill; the remainder from the city of Galveston. There had been three deaths; seven were discharged cured, two remain in the hospital —one approaching convalescence, the other extremely ill; the latter, a woman, who has not been out of Galveston for years shows all the nervous phenomena characteristic of the severest types of typhoid fever—high temperature curves, tremulous dry, brownish and fissured tongue, semi-comatose condition during the last ten days. All of these patients exhibited the successive crops of the rose colored spots, and in the most of them the spleen was enlarged.

Special attention is called to one case, Mr. G., a student from Leon county, as affording a possible explanation of the resistance offered to the effects of quinine shown by certain individuals of the blonde type as pointed out by Dr. Humphreys, of Hawkins, Texas.

Mr. G—— had been in Galveston about two weeks; was taken sick about Oct. 18th with fever, which was supposed to be malarial; he was treated by one of his fellow-students with large and repeated doses of quinia. After two or three days of ineffectual efforts to control the fever with this remedy, I was consulted; knowing the patient came from a malarial country, I advised continuation of the quinia. It was soon found to be entirely useless; the fever marched right along. The alkaloid was stopped; in a few days the characteristic eruption and other pronounced symptoms indicated unmistakably that the fever was typhoid. He was taken to the hospital, where he recovered after a fever lasting about three weeks.

Mr. G—— was a pronounced blonde. This disease resisted the action of quinia, not from any idiosyncrasy, but solely because the fever was enteric and not malarial.

I would like to say in this connection, that my attention having been called to articles in the October and November numbers of the *Courier-Record of Medicine*, by Dr. B. Frank Humphreys, I took the liberty of inviting the Doctor to meet me here, at this meeting, that we might discuss some of the points relating to the subject of fevers in Texas. I received a courteous reply, saying he regretted not being able to get here.

Now, the matter to which I especially wish to direct your attention, and in which I take issue with Dr. H. And others whose sentiments he utters, is this: *They appear to recognize only malarial fever in Texas*, as exemplified in the following quotation from the above mentioned paper (See page 37, *Courier-Record*, Oct.): "It has been observed for several years that our remittents are less easily controlled than formerly, and that their tendency to a continued form is becoming more and more common, which is probably due, in many instances, to neglect or inefficient treatment."

The same idea was expressed more broadly to me a few weeks ago. Incidentally, I asked a brother physician in Galveston, "have you seen any typhoid fever lately? He replied, "No, I have never seen any in Texas, and I don't believe there is any such fever here. Whenever a fever becomes continued, it is the doctor's fault. I know how to use quinine, and always bieak the fever." What reply could I make to such a statement when at that moment I had under treatment at the hospital half a dozen cases of typical typhoid, representative of different portions of the State? While I do not pretend to say that the cases of continued fever observed by Dr. Humphrey, in Hood county, which run for weeks, "completely exhausting the patient," and which are not controlled by quinia, are in reality cases of enteric fever, I will say this, that the extreme probability of this fact should not be ignored.

The sooner the medical profession in this State recognize the reality of the broadcast dissemination of the germs of typhoid fever, the better it will be for them as well as the people. In my opinion, there is no question before the profession of our State to-day that will compare in importance with this. Many cases are no doubt mild ones, and if isolated might be difficult of recognition; but occurring as they undoubtedly do, in the epidemic form, oftentimes affecting several members of the same family, showing identity of ætiology, the nature of such fevers, to my mind, is clear.

The practical importance of this subject is my excuse for again directing attention to the facts going to prove that this fever is enteric, and not malarial, or anything else. 1st. The frequent occurrence of house epidemics, where though most of the cases may be of the mildest type, yet occasionally a case will present the features characteristic of the severest forms of typhoid fever; unfortunately, also, one of the so-called mild cases will every now and then die with symptoms pointing to intestinal perforation, in a household where there may have been other cases, not exhibiting a single unfavorable symptom.

2nd. The very uniform presence of the rose-colored spots, and enlargement of the spleen.

3rd. The fact that these cases are wholly unaffected by the alkaloids of Peruvian bark; that is to say, that the course and duration of the fever will be uncontrolled by quinia, though large doses may temporarily reduce the temperature.

4th. The occasional, not to say frequent supervention of intestinal hemorrhage. The absence of diarrhœa, iliac tenderness, and the severe nervous phenomena is no proof that the fever is typhoid; for these symptoms are just as frequently absent as present.

In reply to Dr. Poynor, concerning the subject of treatment, I will say a few words:

It is unnecessary to discuss the general principles of treatment which are sufficiently elaborated in the text-books. 1st. In regard to the recent and powerful antipyretics belonging to the phenol or coal-tar group. In my opinion, we should be exceedingly cautious in prescribing any of them; the good they can accomplish is very questionable; the evil they may do is certain. They not only are powerful depressants of the nervous and circulatory functions, but modify the condition of the blood itself.

We should be especially guarded with these remedies during the late periods of the fever. Here, cardiac weakness is probably the most important condition we have to combat. I am not making mere theoretical objections, for I have seen very serious depression induced by only seven grains of phenacetin. We have in the use of baths and cool sponging the means by which we may reduce the temperature, and at the same time stimulate the nervous centers, improve the character of the respirations, and in every way benefit the patient.

One word about the use of antiseptics, and I will tax your patience no further:

The theory upon which these remedies are prescribed may be sound; the difficulty is to practice it without detriment to the patient. The rule, I venture to suggest, is whenever any antiseptic has a tendency to produce nausea, or interfere with digestion, it is doing harm, and should be discontinued. The milder agents of this class as preparations of bismuth, pepsine, dilute hydrochloric acid, allay nausea, aid digestion, relieve irritation, and hence may be freely used; but carbolic acid, iodine, naphthol, etc., should be cautiously administered.

